# Prognostic effect of intratumoral neutrophils across histological subtypes of non-small cell lung cancer

**DOI:** 10.18632/oncotarget.12360

**Published:** 2016-09-30

**Authors:** Mehrdad Rakaee, Lill-Tove Busund, Erna-Elise Paulsen, Elin Richardsen, Samer Al-Saad, Sigve Andersen, Tom Donnem, Roy M. Bremnes, Thomas K. Kilvaer

**Affiliations:** ^1^ Department of Medical Biology, University of Tromsø, The Arctic University of Norway, N-9037 Tromso, Norway; ^2^ Department of Clinical Pathology, University Hospital of North Norway, N-9038 Tromso, Norway; ^3^ Department of Clinical Medicine, University of Tromsø, The Arctic University of Norway, N-9037 Tromso, Norway; ^4^ Department of Oncology, University Hospital of North Norway, N-9038 Tromso, Norway

**Keywords:** CD66b, TANs, NSCLC, neutrophils, lung cancer

## Abstract

Recent data indicate that tumor-associated neutrophils (TANs) serve a dual role in tumor progression and regression. CD66b is a neutrophil marker and has been associated with patient outcome in various cancers. However, its clinical impact in non-small cell lung cancer (NSCLC) remains controversial. 536 NSCLC patients, of which 172 harbored lymph node metastases, were included in this study. Tissue microarrays were constructed and multiplexed immunohistochemistry of CD66b, CD34 and pan-keratin was performed to evaluate the localization and quantity of CD66b^+^ TANs. High intratumoral CD66b^+^ TANs density in squamous cell carcinoma (SCC) subgroup was an independent positive prognosticator for disease-specific survival (*P* = 0.038). In contrast, high intratumoral TANs density was an independent negative prognostic factor in the adenocarcinoma (ADC) subgroup (*P*= 0.032). Likewise, in ADC patients with lymph node metastases, high level of intratumoral TANs was associated with poor prognosis (*P* = 0.003). Stromal CD66b^+^ TANs were not associated with outcome of NSCLC patients. In conclusion, CD66b^+^ TANs show diverging prognostic effect in NSCLC patients according to histological subgroups. The presence of CD66b^+^ TANs could prove pivotal for development of an immunoscore in ADC NSCLC patients.

## INTRODUCTION

Lung cancer is the leading cause for cancer-related deaths worldwide [1]. Even after complete surgical resections, the prognosis of NSCLC patients remain poor due to locoregional relapses and/or metastases [2]. Huge efforts are being invested in identifying new prognostic and predictive molecular markers in order to improve treatment stratification and overall survival (OS).

Tumor infiltrating leukocytes (TILs) positively affect NSCLC patient outcomes [3]. Of these, a significant proportion constitute tumor-associated neutrophils (TANs) [4]. It has been hypothesized that TANs polarize into either a N1 antitumoral or N2 protumoral phenotype in response to cancer epithelial- and stromal-derived signals [5, 6]. The protumoral functions of the N2 phenotype include increased angiogenesis [8], tumor cell proliferation [9], extracellular matrix remodelling, lymphangiogenesis and inhibition of the anti-tumoral immune response [10]. The anti-tumoral activity of the N1 phenotype comprises immune-surveillance including cytotoxicity towards cancer cells. The cellular cytotoxicity leads to recruitment and activation of cells related to both the innate and adaptive immune systems [11, 12].

CD66b is an established marker of TANs [13], stored in neutrophil granules and constitutively expressed by human neutrophils [14]. In contrast to the cells of adaptive immune system, the prognostic role of CD66b^+^ TANs has been associated with unfavorable outcome for a number of malignancies [15–18], whereas improved survival has been reported for gastric and colorectal carcinoma [19, 20]. In NSCLC, two previous studies failed to reveal significant associations between TANs and patients outcome [21, 22], neither of these studies evaluated histological subtypes.

Differential outcome of CD66b^+^ TAN presence according to histological subtypes could be expected since squamous cell carcinoma (SCC) and adenocarcinoma (ADC) are recognized as different diseases regarding biology, treatment stratification and efficacy [23]. This harmonizes with our previous studies on the immune contexture in SCC vs ADC NSCLC patients [24].

Herein, we 1) investigate the prognostic role of CD66b^+^ TANs in primary tumors of NSCLC patients stratified into SCC and ADC subgroups, 2) assess the prognostic effect of intraepithelial CD66b^+^ TANs in metastatic lymph nodes from N+ patients and 3) correlate the presence of CD66b^+^ TANs with 104 tumor molecular markers previously evaluated in this same cohort.

## RESULTS

### Patient characteristics

A retrospectively registered cohort of 536 patients with NSCLC, of which 172 had LN metastases at the time of diagnosis, was investigated for CD66b^+^ TAN density. Detailed information of the cohort has previously been published [24, 25]. The average age of the patients at the time of surgery was 67 years old (range, 28-85), and 68% of the patients were men. According to histology, primary tumors were divided into the following histotypes: 289 squamous cell carcinomas (SCC), 201 adenocarcinomas (ADC) and 46 large-cell carcinomas (LCC). Histological features in the LN+ cohort were: 91 (53%) patients SCC, 68 (40%) patients ADC and 13 (8%) patients undifferentiated carcinomas (NOS). During the period 2005-2010, 43 (20%) patients received adjuvant chemotherapy. 76 (14%) patients received adjuvant radiotherapy.

### Interobserver variability

The intraclass correlation coefficients (ICCs) and Cohen's Kappa for the CD66b scores were as follows: Intratumoral primary tumor (ICC = 0.94, *P* < 0.001, Kappa = 0.79, *P* < 0.001), stromal (ICC = 0.93, *P* < 0.001, Kappa = 0.75, *P* < 0.001) and intratumoral lymph node metastases (ICC = 0.95, *P* < 0.001, Kappa = 0.80, *P* < 0.001).

### Expression and association of CD66b^+^ TANs with histopathological variables

**Figure 1:**
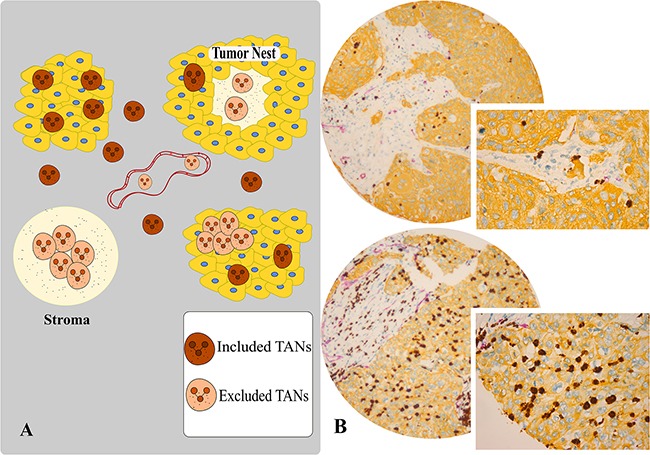
**A.** Scoring assessment guideline. Scoring of intratumoral (tumor nest) and stromal CD66b^+^ neutrophils was conducted utilizing the following exclusion- and inclusion-criteria. In intratumoral assessment, we have included only unaggregated CD66b^+^ cells completely surrounded by tumor epithelial cells. Stromal assessment only regarded extravascular CD66b^+^ cells. The excluded areas consisted of intratumoral and stromal aggregates of neutrophils, intravascular neutrophils and CD66b^+^ cells with granular background in stroma. Especially in SCC tissue, the central tumor zone often had dense granular CD66^+^ structures with some CD66b^+^ cells, considered a pre- or necrotic area, which were excluded from scoring. **B.** Multiplexed IHC analysis of TANs with CD66b/CD34/pan-CK panel, high versus low intratumoral densities. Brown, purple and yellow substrates were applied to visualize CD66b, CD34 and pan-CK respectively (magnification 10x, 20x).

CD66b was expressed on the membrane and in the cytoplasm of TANs, while multiplex IHC allowed effortless distinction between intratumoral, stromal and intra-vascular CD66^+^ TANs (Figure 1). Associations between CD66b^+^ TAN density and clinicopathological variables in the overall cohort (primary tumor, LN+) and within histological subtypes are presented in Table 1. Intratumoral CD66b^+^ TANs in the primary tumor were positively associated with increasing tStage (*P* = 0.011) and pStage IIB (*P* = 0.002) in the whole cohort, and was negatively associated with nStage (*P* = 0.032) in the SCC subgroup. Stromal CD66b^+^ cells were associated with weight loss in the whole cohort (*P* = 0.044) and in the SCC subgroup (*P* = 0.031). No associations between clinicopathological variables and the presence of CD66b^+^ TANs were observed in the ADC-group or in the N+ subgroups (Table 1).

**Table 1:** Correlations between clinicopathological variables and A) Intratumoral CD66b^+^ cells in resected primary tumors of NSCLC patients in the overall cohort and stratified into the SCC and ADC subgroups (chi-square test and fisher exact test, N = 536, 289 and 201 respectively); B) Intratumoral CD66b^+^ cells in lymph-node tissue from N+ NSCLC patients in the overall cohort and stratified into the SCC and ADC subgroups (chi-square test and fisher exact test, N = 172, 91 and 68 respectively)

	A									B								
	All			SCC			ADC			All			SCC			ADC		
	Low	High	P	Low	High	P	Low	High	P	Low	High	P	Low	High	P	Low	High	P
Age			0.535			0.223			0.023			1.000			0.284			0.367
≤65	129	85		65	38		54	39		41	17		22	6		18	10	
>65	187	108		96	79		71	24		42	17		18	11		17	5	
Gender			0.221			0.158			0.522			0.130			0.706			0.069
Female	92	67		35	35		48	28		28	6		8	2		17	3	
Male	224	126		126	82		77	35		55	28		32	15		18	12	
ECOG			0.092			0.189			0.394			0.407			0.432			1.000
0	185	113		88	65		75	42		48	15		22	6		20	9	
1	115	61		65	40		43	16		28	15		15	9		11	5	
2	16	19		8	12		7	5		7	4		3	2		4	1	
Smoking			0.376			1.000			0.497			0.931			0.299			0.175
Never	12	3		4	2		7	1		3	1		2	0		1	1	
Previous	200	124		101	74		77	40		49	21		25	8		20	12	
Present	104	66		54	41		41	22		31	12		13	9		14	2	
Weight loss			0.061			0.183			1.000			0.195			0.058			0.654
<10%	291	167		147	100		116	58		76	28		38	13		30	14	
≥10%	25	25		14	17		9	5		7	6		2	4		5	1	
Surgical procedure			1.000			0.276			0.867			0.703			1.000			0.746
Wedge/Lobectomy	229	140		103	83		100	49		44	16		16	7		24	9	
Pulmonectomy	87	53		58	34		25	14		39	18		24	10		11	6	
Margins			0.357			0.031			1.000			0.131			0.428			0.152
Free	286	180		138	110		118	60		75	27		35	13		33	12	
Not free	30	13		23	7		7	3		8	7		5	4		2	3	
tStage			0.011			0.119			0.431			0.064			0.495			0.371
IA	48	22		23	13		23	9		6	1		3	1		3	0	
IB	60	30		27	19		27	10		15	1		5	0		7	1	
IIA	120	58		57	30		50	23		31	13		15	6		15	7	
IIB	43	30		29	22		10	7		12	6		7	5		4	1	
III	42	50		23	32		14	13		15	13		7	5		6	6	
IV	3	3		2	1		1	1		4	0		3	0				
nStage			0.072			0.032			0.607			0.584			0.315			1.000
0	204	143		101	90		81	45										
1	76	36		46	23		27	10		55	25		30	15		21	9	
2	36	14		14	4		17	8		28	9		10	2		14	6	
pStage			0.002			0.061			0.070			0.229			0.386			0.548
IA	81	47		36	30		39	17										
IB	70	44		32	23		28	16										
IIA	82	33		50	25		26	7		36	10		19	6		15	4	
IIB	30	41		19	27		8	12		6	5		4	4		2	1	
IIIA	53	28		24	12		24	11		41	19		17	7		18	10	
Histology			0.096									0.908						
SCC	161	117								40	17							
ADC	125	63								35	15							
NOS	30	13								8	2							
Differentiation			0.674			0.851			0.083			0.655			0.794			0.236
Poor	132	84		58	40		44	31		41	20		15	7		18	11	
Moderate	143	89		87	63		56	26		37	13		22	10		15	3	
Well	41	20		16	14		25	6		5	1		3	0		2	1	
Vascular infiltration			0.493			0.218			1.000			0.686			0.752			1.000
No	253	161		124	98		106	54		58	25		27	13		27	11	
Yes	61	32		37	19		17	9		25	8		13	4		8	3	

Abbreviations: SCC, squamous cell carcinoma; ADC, adenocarcinoma; ECOG, Eastern Cooperative Oncology Group; NOS, not otherwise specified

### Survival analyses

**Figure 2:**
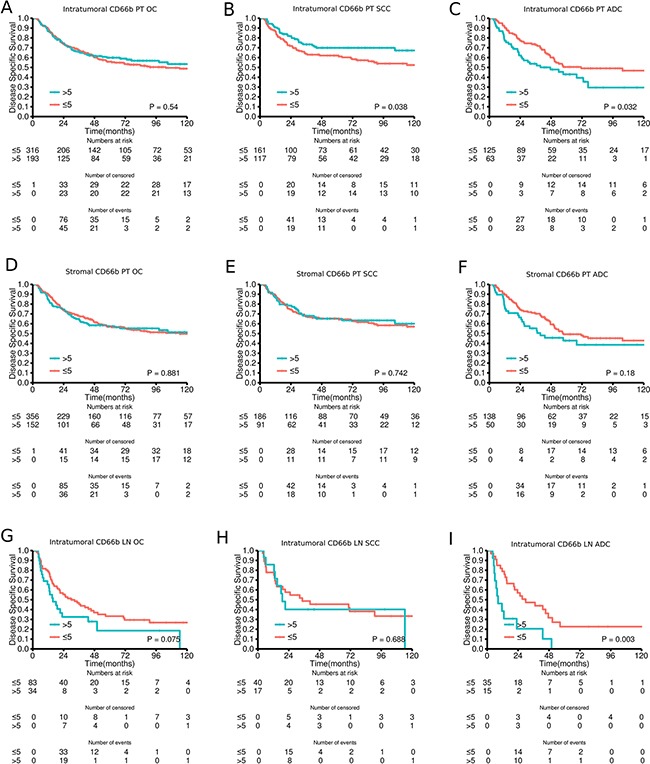
Disease-specific survival curves for **A.** Intratumoral CD66b in the overall cohort (OC) of primary tumors (PT); **B.** Intratumoral CD66b in squamous cell carcinomas (SCC) of PT; **C.** Intratumoral CD66b in adenocarcinomas (ADC) of PT; **D.** Stromal CD66b in the overall cohort of PT; **E.** Stromal CD66b in SCC of PT; **F.** Stromal CD66b in ADC of PT; **G.** Intratumoral CD66b in the overall cohort of LN+; **H.** Intratumoral CD66b in SCC of LN+; **I.** Intratumoral CD66b in ADC of LN+

Tables 2 and 3 and Figure 2 summarize the intratumoral presence of CD66b^+^ cells in primary tumors and lymph node metastases and their role on diseases-specific survival (DSS) in univariable and multivariable analyses. No significant effects on survival were observed in the overall cohort. In the SCC subgroup, the presence of intratumoral CD66b^+^ cells was an independent positive prognostic factor [univariate *P* = 0.038 (Figure 2B), multivariable *P* = 0.021, HR 0.59 (0.38-0.92)], while in the ADC subgroup, the presence of intratumoral CD66b^+^ cells was an independent adverse prognostic factor [univariate *P* = 0.032 (Figure 2C), multivariable *P* = 0.020, HR 1.7 (1.1-2.65)]. In patients with nodal metastases (Figure 2G–2I), the presence of intratumoral CD66b^+^ cells was an independent adverse prognostic factor for the ADC subgroup [univariate *P* = 0.003 (Figure 2I), multivariable *P* = 0.004, HR = 2.87 (1.39-5.91)]. Results were largely similar when assessing disease-free survival (DFS) and OS (Supplementary Figures S1 and S2). The density of CD66^+^ neutrophils in the stroma was not significantly associated with survival (Figure 2D–2F).

**Table 2:** **A)** Intratumoral CD66b^+^ cells in the primary tumors of resected NSCLC patients as predictors of disease-specific survival in the overall cohort and stratified into SCC and ADC subgroups (univariate analyses, log-rank test, N = 536, 289 and 201 respectively). **B)** Intratumoral CD66b^+^ cells in lymph-node tissue from N+ NSCLC patients as predictors of disease-specific survival in the overall cohort and stratified into SCC and ADC subgroups (univariate analyses, log-rank test, N = 172, 91 and 68 respectively)

	All					SCC					ADC				
A	N(%)	5 Year	Median	HR (95%CI)	P	N(%)	5 Year	Median	HR (95%CI)	P	N(%)	5 Year	Median	HR (95%CI)	P
CD66b Primary tumor					0.540					0.038					0.032
≤5%	316(59)	56	104	1		161(56)	62	235	1		125(62)	50	71	1	
>5%	193(36)	60	NA	0.92 (0.69-1.21)		117(40)	70	NA	0.64 (0.43-0.96)		63(31)	43	47	1.57 (1-2.46)	
missing	27(5)					11(4)					13(6)				
**B**															
CD66b LN+					0.075					0.688					0.003
≤5%	83(48)	33	30	1		40(44)	45	35	1		35(51)	23	30	1	
>5%	34(20)	19	16	1.57 (0.9-2.74)		17(19)	40	19	1.17 (0.52-2.61)		15(22)	0	8	2.71 (1.1-6.65)	
missing	55(32)					34(37)					18(26)				

Abbreviations: SCC, squamous cell carcinoma; ADC, adenocarcinoma; LN+, metastatic lymph nodes

**Table 3:** Multivariable models summarizing significant independent prognostic factors in A) The SCC and ADC subgroups of the total cohort (Cox regression analyses, N = 289 and 201 respectively) and B) The ADC subgroup of the N+ NSCLC patients (Cox regression analyses, N = 68)

	A	Overall cohort of NSCLC patients	SCC		ADC		B	NSCLC patients with N+ cohort	ADC	
			HR (95% CI)	P	HR (95% CI)	P			HR (95% CI)	P
Intratumoral CD66b										
≤5%			1.000		1.000				1.000	
>5%			0.59 (0.38-0.92)	**0.021**	1.7 (1.1-2.65)	**0.020**			2.87 (1.39-5.91)	**0.004**
pStage										
IA			1.000		1.000					
IB			1.17 (0.51-2.71)	0.715	1.93 (1.02-3.64)	**0.043**				
IIA			2.09 (1.05-4.17)	**0.036**	3.07 (1.58-5.97)	**<0.001**				
IIB			4.43 (2.19-8.94)	**<0.001**	2.51 (1.18-5.35)	**0.017**				
IIIA			7.98 (3.97-16.03)	**<0.001**	4.62 (2.38-8.97)	**<0.001**				
Different										
Poor					1.000					
Moderate					0.91 (0.59-1.42)	0.682				
Well					0.44 (0.22-0.89)	**0.022**				
ECOG										
0					1.000					
1					1.76 (1.13-2.74)	**0.012**				
2					2.93 (1.28-6.7)	**0.011**				

Abbreviations: SCC, squamous cell carcinoma; ADC, adenocarcinoma; ECOG, Eastern Cooperative Oncology Group

### CD66b^+^ TANs and treatment interactions

The adjuvant chemotherapy treatment-TAN interaction was not significant for OS, DFS and DSS in either overall cohort or ADC and SCC subgroups (data not shown).

Further, for patients who received adjuvant radiotherapy, there was a tendency towards increased survival differences between patients with high vs low CD66b density. Although, similar tendencies were seen for patients who did not receive radiotherapy, and subgroup analysis should be interpreted with caution because of the small sample size [SCC: n=41; ADC: n=19] (data not shown).

### Presence of CD66b^+^ neutrophils and their correlations with prognostic markers

Table 4 summarizes the significant correlations between the presence of CD66b^+^ cells and prognostic markers previously studied in this cohort. Supplementary Table S1 includes all markers involved in the correlation analysis (n=104). After corrections for multiple testing, intratumoral CD66b^+^ cells in the whole cohort, were correlated with stromal CD66b^+^ (*r* = 0.76), CSF1R (*r* = 0.23), MCSF (*r* = 0.28), MCT1 (*r* = 0.23) and MCT4 (*r* = 0.22) and intratumoral CD68^+^ cells (*r* = 0.23). Stromal CD66b^+^ cells were correlated with intratumoral CD66b^+^ (*r* = 0.76) and stromal CSF1R (*r* = 0.25), CD68^+^ cells (*r* = 0.23), MCSF (*r* = 0.25) and MCT1 (*r* = 0.26). In the SCC subgroup, intratumoral CD66b^+^ cells were correlated with stromal expression of CD66b^+^ (*r* = 0.80) and MCSF (*r* = 0.32), while stromal CD66b^+^ cells were correlated with intratumoral CD66b^+^ (*r* = 0.80), CD68^+^ cells (*r* = 0.28), MCT4 (*r* = 0.27) and stromal CSF1R (*r* = 0.28), stromal CD68^+^ cells (*r* = 0.27), MCSF (*r* = 0.32), FOXO1A (*r* = 0.28) and MCT1 (*r* = 0.30). In the ADC subgroup intratumoral CD66b^+^ cells correlated only with stromal CD66b^+^ cells (*r* = 0.66).

**Table 4:** Significant Spearman rank-correlations with *R*-values > 0.20 between intratumoral and stromal CD66b^+^ TANs and tumor-associated markers in samples from NSCLC in the total cohort and in subgroups according to histology (Total cohort = 326, SCC = 191; ADC = 95)

	ALL		SCC		ADC			
	Correlations with markers expressed in intratumoral cells							
	Tumor	Stroma	Tumor	Stroma	Tumor	Stroma		
CAIX						0.23*	ANG	T
CD34					−0.27**	−0.27**	ANG	T
D240					0.21*		ANG	T
DLL4	−0.20***					−0.24*	ANG	T
FGFR1					−0.23*		ANG	T
Glut1					0.28**		ANG	T
NOTCH1					0.25*		ANG	T
NOTCH4			−0.22**				ANG	T
PHD-3						−0.33***	ANG	T
Bad-cyt			−0.24***		−0.21*	−0.22*	EMT	T
Bad-Nuc			−0.24**				EMT	T
Her3			−0.21**				EMT	T
Ki67					0.22*		EMT	T
pHer2				−0.22**			EMT	T
pi3K			−0.21***				EMT	T
CD66b		0.76#		0.80#		0.66#	IMM	T
CD68	0.23#		0.28***	0.28#			IMM	T
MCT1						0.22*	MET	T
MCT4		0.22#	0.22**	0.27#			MET	T
PGC1-α						−0.23*	MET	T
	**Correlations with markers expressed in tumor stroma**							
	**Tumor**	**Stroma**	**Tumor**	**Stroma**	**Tumor**	**Stroma**		
Ang2					0.21*		ANG	S
D240					0.24*		ANG	S
DLL4				0.21**			ANG	S
miR21		0.21***				0.23*	ANG	S
NOTCH4				0.21**			ANG	S
PDGF-A			0.22**	0.23**	0.23*		ANG	S
VEGF-A			0.22**				ANG	S
VEGF-D				0.21**	0.21*		ANG	S
cAkt				0.24***			EMT	S
ERK3				0.20**			EMT	S
IGF1				0.21**			EMT	S
NfκB				0.23**			EMT	S
PAR6			0.22**				EMT	S
CD138				0.21**			IMM	S
CD1a			0.20**				IMM	S
CD66b	0.76#		0.80#		0.66#		IMM	S
CSF1R	0.23#	0.25#	0.26***	0.28#	0.23*	0.21*	IMM	S
CXCL16				0.25***			IMM	S
FOXP3				0.21**			IMM	S
CD68		0.23#	0.23**	0.27#			IMM	S
MCSF	0.28#	0.25#	0.32#	0.32#			IMM	S
FOXO1A				0.28#			IMM	S
LDH5				0.24***			MET	S
MCT1	0.23#	0.26#	0.23**	0.30#	0.28**	0.24*	MET	S
MCT4	0.22#	0.20***	0.20**		0.23*	0.27**	MET	S

Abbreviations: CD, cluster of differentiation; SCC, squamous cell carcinoma; ADC, adenocarcinoma;ANG, angiogenesis; EMT, epithelial-mesenchymal transition; MET, metastasis; IMM; immunology; CAIX, Anti-Carbonic Anhydrase IX;DLL4, Delta ligand 4; FGFR1, Fibroblast Growth Factor Receptor 1; GLUT1, Glucose transporter 1; PHD, prolyl hydroxylase-domain; BAD, Bcl2 Associated Death Promoter; Cyt, in cytoplasm; Nuc, in nucleus; Her, human epidermal growth factor receptor; MCT, monocarboxylate transporter; PGC1, Peroxisome proliferative activated receptor gamma coactivator 1; Ang, angiogenin; miR, micro RNA; PDGF, Platelet-derived growth factor; VEGF, Vascular endothelial growth factor; IGF1, Insulin like growth factor 1; PAR6, Partitioning defective 6; CXCL16, C-X-C motif ligand; FOX, forkhead box; MCSF, Macrophage Colony Stimulating Factor 1; CSF1R, Colony stimulating factor 1 receptor; LDH, lactate dehydrogenase.

* significant at p > 0.05,

** significant at p > 0.01,

*** significant at p >0.001,

# significant after Bonferroni correction for multiple tests

## DISCUSSION

In our large cohort of unselected stage I-IIIA NSCLC patients, we demonstrate that the presence of intratumoral CD66b^+^ neutrophils mediate opposing independent prognostic significance in the ADC versus SCC subtype. Moreover, this prognostic significance goes undetected when the role of TANs is investigated in the whole NSCLC cohort, and not according to histology. Lymph nodes from LN+ patients of the same cohort, when evaluated as a validation cohort, revealed similar prognostic harmony with their corresponding primary tumors.

CD66b is recognized as a TAN marker in several studies, and a recent meta-analysis reported the presence of CD66b^+^ TANs to be a significant unfavorable prognosticator in solid malignant tumors [26]. In NSCLC, the role of CD66b^+^ TANs remains controversial. Ilie et al. [22] found the CD66b^+^ TAN/CD8^+^ T cell ratio to predict recurrence and poor OS. In contrast Carus et al. [21] did not detect an association between CD66b^+^ TANs and survival. Nevertheless, studies in renal cell [15], head and neck [16], bronchioloalveolar [17] and oesophageal carcinoma [18] have demonstrated association between the presence of intratumoral CD66b^+^ neutrophils and poor prognosis, while in gastric and colorectal carcinoma high levels of CD66b^+^ neutrophils indicated a favorable prognosis [19, 20].

The current knowledge of TAN function is based on studies in tumor-bearing animal models, not in humans. Deletion or alteration of TGF-β signaling within the tumor lead to reduced tumor progression through the activation of CD8^+^ T cells and recruitment of myeloid-derived suppressor cells (MDSCs) [27]. In a previous *in vivo* study, TGF- β signaling observed in tumor-bearing mice exerted effects on polymorphonuclear lineages of MDSCs and induced a distinct N2 TAN subtype, which promoted cancer development. Furthermore, abrogation of TGF- β signaling polarized TANs from the protumoral N2 to the antitumoral N1 phenotype. Depletion of antitumoral N1 TANs following TGF-β blockade reduced CD8^+^ T cells activation and promoted tumor growth [5]. In a mouse model, N1 TAN mediated activation of CD8^+^ T cells has been determined the main mechanism responsible for mediating an antitumoral response. It is tempting to infer that the same mechanisms are present in humans, but as with M1/M2 tissue macrophage differentiation, there are broad differences between tumor-bearing mice and humans. As macrophages and neutrophils ascend from a common progenitor, the complexity observed in human macrophage differentiation should be expected for neutrophils as well [28]. Not surprisingly, we observe a close correlation between CD66b^+^ TANs and CD68^+^ (pan-macrophage marker) expression in our cohort (Table 4) and the MDSC associated colony-stimulating factor-1 receptor, indicating a close relationship between TANs and macrophages in NSCLC.

The idea that human TANs may differentially affect the tumor-host immune activity based on stage and histological subtype of cancer is intriguing. In early stage NSCLC, TANs have been shown not to be mainly immunosuppressive, but would rather stimulate T cell-mediated immunity through the production of co-stimulatory molecules enhancing proliferation of CD4^+^and CD8^+^ T cells [29, 30]. The role of human TANs in perturbing immunity is poorly defined mechanistically, and gaps remain in understanding TANs plasticity and the switch between pro- and antitumoral effects *in vitro*. Further, little is known of the *in vivo* properties of TANs and whether *in vitro* data from studies in mice can be extrapolated to humans. Moreover, a plethora of signaling molecules, differing between and even within different stages of the same histological subtypes, are available to influence TANs. This is a plausible explanation for why the prognostic significance of CD66b^+^ TANs diverging according to histological subtypes. This is supported by the two predominant NSCLC histological phenotypes, SCC and ADC, displaying distinct differences in genomic and stromal heterogeneity, association to smoking, growth pattern and sensitivity to treatment [23], and are by many regarded as different entities.

Tumor infiltrating immune cells have a pivotal contribution in cancer progression and critically influences the clinical outcome of patients depending on density and localization of various immune cell subsets [31]. The analysis of the immune contexture in NSCLC has revealed supplementary prognostic and predictive data which may be combined with the standard pathological TNM classification to form a TNM-Immunoscore (TNM-I) [32]. Recently, our group have reported stromal CD8^+^ and CD45RO^+^ TILs to positively associate with survival, hence being good candidate markers for a TNM-I in NSCLC [24, 33]. The effect of TILs in NSCLC is best documented in the SCC subgroup, while the data presented herein indicate that CD66b^+^ TANs to be a candidate in TNM-I for the ADC subgroup. Supplementary Figure S3 shows how incorporation of CD66^+^ TAN status could improve the prognostic properties of the established TNM staging system for NSCLC ADC patients. However, these data are preliminary and need to be confirmed in larger NSCLC ADC cohorts.

In conclusion, we observed that intratumoral CD66b^+^ TANs is both an independent positive and negative prognosticator in the SCC and ADC subgroup of NSCLC patients, respectively. While CD8^+^/CD3^+^/CD45RO^+^ TILs seem to be pivotal for the establishment of a TNM-I for NSCLC SCC patients, CD66b^+^ TANs may prove an appropriate choice for ADC patients.

## MATERIALS AND METHODS

### Patients and ethical clearance

An unselected population of 536 patients with surgically resected stage I-IIIA NSCLC from the University Hospital of North-Norway and Nordland Central Hospital from 1990-2010 were included in this study. Of 536 patients, 509 were involved in survival analysis, while the remaining cases are highlighted as missing due to poor tissue quality and unscorable cores.

For the N+ patients, the total number (n=172) constitutes all patients in the cohort with N+ disease. Of these 55 were either missing due to bad or missing cores or due the fact that LN tissue was not available in the archival tissue. Both study populations are described previously by our group [24, 25].

The study was approved by the Regional Committee for Medical and Health Research Ethics (Northern Norway, UNN: protocol ID: 2011/2503). Data collection and storing of the clinical database were approved by the National Data Inspection Board. The study instructions for biomarker expression, clinicopathological features and survival data is reported according to the REMARK guidelines [34].

### Tissue micro-array construction and Immunohistochemistry

All tissue samples were reviewed by two experienced pathologists (ER.LTB). The most representative areas was marked on the hemotoxylin and eosin (H&E) slide and sampled for tissue micro-array (TMA) blocks. The TMAs were assembled using a tissue-arraying instrument (Beecher Instruments, Silver Springs, MD, USA). The methodology has previously been explained [35]. Briefly, four 0.6 mm cores were sampled for each patient: two from central tumor epithelium and two from tumor stroma.

### Multiplexed Immunohistochemistry

Triple IHC staining was carried out sequentially using the Discovery-Ultra automated immunostainer (Ventana Medical Systems, Tucson, AZ). Deparaffinization and on-board antigen retrieval were performed for 32 minutes at approximately 100°C with CC1 reagent, which is an EDTA-based proprietary Ventana solution (pH 8.0–8.5). CD66b mouse monoclonal antibody (#555723, clone:G10F5, BD Biosciences, dilution 1/400) was applied and incubated for 32 min and amplified for 4 min, followed by an ultraWash step to wash off excess antibody. Antibody denaturation for 8 minutes at 90°C was performed to ensure that the first primary antibody was completely inactivated before applying the second antibody. The pre-diluted CD34 mouse monoclonal antibody (#790-2927, Ventana, Clone: QBEnd/10) was then applied as a second primary antibody and was incubated for 32 minutes, and then washed, followed by denaturation. In the last sequence pre-diluted mouse monoclonal anti-pan keratin (CK) (#760-2135, Ventana, Clone: AE1/AE3/PCK26) was applied with 16 min incubation. The primary antibodies CD66b, CD34 and pan CK were visualized using Ventana DAB, Purple, Yellow detection kits respectively with 32 min incubation time for DAB, 16 min for purple and 1 hour for yellow chromogens. Finally, the slides were counterstained with hematoxylin and bluing reagent.

All triple stained sections were compared with the corresponding single stained section slide. Three different controls for our staining method were applied: 1) A blank control by omission of the primary antibody in every sequence of staining, 2) control staining of the sections with an isotype-matched control antibody without the primary antibody and 3) multiple organ TMAs as positive and negative tissue controls to verify the specificity of the staining for every staining procedure

### Scoring of immunohistochemistry

Two pathologists (LTB, ER) established a semi-quantitative score. Pan-keratin identified normal and malignant epithelium, while CD34 differentiated intra- and extra-vascular CD66b^+^ TANs.

The TMA slides were scored by two observers (MR, EEP) for intratumoral (primary tumors and lymph nodes) and stromal (primary tumors) CD66b^+^ TANs. A four-tiered scale with the following levels: 0 ≤ 1, 1 = 1-5, 2 = 6-15, 3 >15 for both the tumor epithelial and stromal compartments was used. Intravascular CD66b^+^ TANs, necrotic and pre-necrotic areas were disregarded. The method used for pathological evaluation of CD66b^+^ neutrophil density in tumor and stromal compartments is presented in Figure 1A. Representative images of triple staining with low and high densities are shown in Figure 1B.

For statistical analysis, dichotomization was done and high presence defined as >5 CD66b^+^ neutrophils (score 2 or 3).

### TMA Validation

Whole-tissue section slides of 20 patients were evaluated for tumor heterogeneity with corresponding TMA cores. The selected cases was from different histological and pathological stages with following detail: 10 specimen of ADC (5 tStage I, 5 tStage III), 10 specimen of SCC (5 tStage I, 5 tStage III). The applied staining procedure for whole-tissue section was the same as for TMA slides. Heterogeneity between paired TMA core and whole tissue was low and a significant concordance was observed for TAN density intratumorally (paired *T*-test correlation= 0.91; *p* = <0.001).

### Statistical methods

All data analyses were conducted in RStudio with R version 3.2.2 utilizing the packages survival, gridExtra, car, Hmisc, irr and ggplot2.

IHC scores were compared for interobserver reliability using a two-way random effects model with an absolute agreement definition and Cohen's kappa-statistics with equal weights. The intraclass correlation coefficient and Cohen's kappa were obtained from these results.

The Chi-square and Fischer's Exact tests were used to examine the association between marker expression and clinicopathological variables. Spearman’s rank-correlation was used to examine the associations between marker expressions. Due to the large number variables analyzed in the correlation analyses, Bonferroni corrections were conducted for these analyses.

Univariate survival analyses, were done using the Kaplan-Meier method and statistical difference between survival curves assessed by the log-rank test. DSS, DFS and OS were used as end-points. Multivariable analysis, using the Cox proportional hazards model, was carried out to assess the independent value of pretreatment variables in the presence of other variables. Only variables with *P* < 0.25 from the univariate analyses or otherwise deemed important were explored in multivariable analyses. The significance level used for survival analyses was *P* < 0.05.

## SUPPLEMENTARY FIGURES AND TABLES





## References

[1] Torre LA, Bray F, Siegel RL, Ferlay J, Lortet-tieulent J, Jemal A (2015). Global Cancer Statistics, 2012. CA a cancer J Clin.

[2] Edwards BK, Noone A-M, Mariotto AB, Simard EP, Boscoe FP, Henley SJ, Jemal A, Cho H, Anderson RN, Kohler BA, Eheman CR, Ward EM (2014). Annual Report to the Nation on the status of cancer, 1975-2010, featuring prevalence of comorbidity and impact on survival among persons with lung, colorectal, breast, or prostate cancer. Cancer.

[3] Remark R, Becker C, Gomez JE, Damotte D, Dieu-Nosjean M-C, Sautès-Fridman C, Fridman W-H, Powell C a, Altorki NK, Merad M, Gnjatic S (2015). The Non–Small Cell Lung Cancer Immune Contexture. A Major Determinant of Tumor Characteristics and Patient Outcome. Am J Respir Crit Care Med.

[4] Gregory a D, McGarry Houghton a (2011). Tumor-Associated Neutrophils: New Targets for Cancer Therapy. Cancer Res.

[5] Fridlender ZG, Sun J, Kim S, Kapoor V, Cheng G, Ling L, Worthen GS, Albelda SM (2009). Polarization of tumor-associated neutrophil phenotype by TGF-beta: “N1” versus “N2” TAN. Cancer Cell.

[6] Powell DR, Huttenlocher A (2015). Neutrophils in the Tumor Microenvironment. Trends Immunol.

[7] Nozawa H, Chiu C, Hanahan D (2006). Infiltrating neutrophils mediate the initial angiogenic switch in a mouse model of multistage carcinogenesis. Proc Natl Acad Sci U S A.

[8] Huh SJ, Liang S, Sharma A, Dong C, Robertson GP (2010). Transiently entrapped circulating tumor cells interact with neutrophils to facilitate lung metastasis development. Cancer Res.

[9] Houghton AM, Rzymkiewicz DM, Ji H, Gregory AD, Egea EE, Metz HE, Stolz DB, Land SR, Marconcini LA, Kliment CR, Jenkins KM, Beaulieu KA, Mouded M (2010). Neutrophil elastase-mediated degradation of IRS-1 accelerates lung tumor growth. Nat Med.

[10] Mantovani A, Mantovani A, Allavena P, Allavena P, Sica A, Sica A, Balkwill F, Balkwill F (2008). Cancer-related inflammation. Nature.

[11] Di Carlo E, Forni G, Lollini P, Colombo MP, Modesti A, Musiani P (2001). The intriguing role of polymorphonuclear neutrophils in antitumor reactions. Blood.

[12] Tecchio C, Scapini P, Pizzolo G, Cassatella M a (2013). On the cytokines produced by human neutrophils in tumors. Semin Cancer Biol.

[13] Zhao L, Xu S, Fjaertoft G, Pauksen K, Håkansson L, Venge P (2004). An enzyme-linked immunosorbent assay for human carcinoembryonic antigen-related cell adhesion molecule 8, a biological marker of granulocyte activities in vivo. J Immunol Methods.

[14] Lominadze G, Powell DW, Luerman GC, Link AJ, Ward R a, McLeish KR (2005). Proteomic Analysis of Human Neutrophil Granules. Mol Cell proteomics.

[15] Jensen HK, Donskov F, Marcussen N, Nordsmark M, Lundbeck F, Von Der Maase H (2009). Presence of intratumoral neutrophils is an independent prognostic factor in localized renal cell carcinoma. J Clin Oncol.

[16] Trellakis S, Bruderek K, Dumitru C a, Gholaman H, Gu X, Bankfalvi A, Scherag A, Hütte J, Dominas N, Lehnerdt GF, Hoffmann TK, Lang S, Brandau S (2011). Polymorphonuclear granulocytes in human head and neck cancer: Enhanced inflammatory activity, modulation by cancer cells and expansion in advanced disease. Int J Cancer.

[17] Wislez M, Rabbe N, Marchal J, Milleron B, Crestani B, Mayaud C, Antoine M, Soler P, Cadranel J (2003). Hepatocyte growth factor production by neutrophils infiltrating bronchioloalveolar subtype pulmonary adenocarcinoma: role in tumor progression and death. Cancer Res.

[18] Wang J, Jia Y, Wang N, Zhang X, Tan B, Zhang G, Cheng Y (2014). The clinical significance of tumor-infiltrating neutrophils and neutrophil-to-CD8+ lymphocyte ratio in patients with resectable esophageal squamous cell carcinoma. J Transl Med.

[19] Caruso RA, Bellocco R, Pagano M, Bertoli G, Rigoli L, Inferrera C (2002). Prognostic value of intratumoral neutrophils in advanced gastric carcinoma in a high-risk area in northern Italy. Mod Pathol.

[20] Galdiero MR, Bianchi P, Grizzi F, Di Caro G, Basso G, Ponzetta A, Bonavita E, Barbagallo M, Tartari S, Polentarutti N, Malesci A, Marone G, Roncalli M, Laghi L, Garlanda C, Mantovani A, Jaillon S (2016). Occurrence and significance of tumor-associated neutrophils in patients with colorectal cancer. Int J Cancer.

[21] Carus A, Ladekarl M, Hager H, Pilegaard H, Nielsen PS, Donskov F (2013). Tumor-associated neutrophils and macrophages in non-small cell lung cancer: no immediate impact on patient outcome. Lung Cancer.

[22] Ilie M, Hofman V, Ortholan C, Bonnetaud C, Coëlle C, Mouroux J, Hofman P (2012). Predictive clinical outcome of the intratumoral CD66b-positive neutrophil-to-CD8-positive T-cell ratio in patients with resectable nonsmall cell lung cancer. Cancer.

[23] Chen Z, Fillmore CM, Hammerman PS, Kim CF, Wong K-K (2014). Non-small-cell lung cancers: a heterogeneous set of diseases. Nat Rev Cancer.

[24] Paulsen E-E, Kilvaer T, Khanehkenari MR, Maurseth RJ, Al-Saad S, Hald SM, Al-Shibli K, Andersen S, Richardsen E, Busund L-T, Bremnes R, Donnem T (2015). CD45RO+ Memory T Lymphocytes — a Candidate Marker for TNM-Immunoscore in Squamous Non–Small Cell Lung Cancer. Neoplasia.

[25] Kilvaer TK, Paulsen E, Khanehkenari MR, Al-Saad S, Johansen RM, Al-Shibli K, Bremnes RM, Busund L, Donnem T (2016). The presence of intraepithelial CD45RO+ cells in resected lymph nodes with metastases from NSCLC patients is an independent predictor of disease-specific survival. Br J Cancer.

[26] Shen M, Hu P, Donskov F, Wang G, Liu Q, Du J (2014). Tumor-associated neutrophils as a new prognostic factor in cancer: a systematic review and meta-analysis. PLoS One.

[27] Yang L, Huang J, Ren X, Gorska AE, Chytil A, Aakre M, Carbone DP, Matrisian LM, Richmond A, Lin PC, Moses HL (2008). Abrogation of TGFβ Signaling in Mammary Carcinomas Recruits Gr-1+CD11b+ Myeloid Cells that Promote Metastasis. Cancer Cell.

[28] Mantovani A (2009). The Yin-Yang of Tumor-Associated Neutrophils. Cancer Cell.

[29] Eruslanov EB, Bhojnagarwala PS, Quatromoni JG, Stephen TL, Ranganathan A, Deshpande C, Akimova T, Vachani A, Litzky L, Hancock WW, Conejo-garcia JR, Feldman M, Albelda SM (2014). Tumor-associated neutrophils stimulate T cell resposnses in early-stage human lung cancer. J Clin Invest.

[30] Singhal S, Bhojnagarwala PS, O'Brien S, Moon EK, Garfall AL, Rao AS, Quatromoni JG, Stephen TL, Litzky L, Deshpande C, Feldman MD, Hancock WW, Conejo-Garcia JR (2016). Origin and Role of a Subset of Tumor-Associated Neutrophils with Antigen-Presenting Cell Features in Early-Stage Human Lung Cancer. Cancer Cell.

[31] Fridman WH, Pagès F, Sautès-Fridman C, Galon J (2012). The immune contexture in human tumours: impact on clinical outcome. Nat Rev Cancer.

[32] Donnem T, Kilvaer TK, Andersen S, Richardsen E, Paulsen EE, Hald SM, Al-Saad S, Brustugun OT, Helland A, Lund-Iversen M, Solberg S, Gronberg BH, Wahl SGF (2016). Strategies for clinical implementation of TNM-Immunoscore in resected nonsmall-cell lung cancer. Ann Oncol.

[33] Donnem T, Hald SM, Paulsen E-E, Richardsen E, Al-Saad S, Kilvaer TK, Brustugun OT, Helland A, Lund-Iversen M, Poehl M, Olsen KE, Ditzel HJ, Hansen O (2015). Stromal CD8+ T-cell Density—A Promising Supplement to TNM Staging in Non-Small Cell Lung Cancer. Clin Cancer Res.

[34] McShane LM, Altman DG, Sauerbrei W, Taube SE, Gion M, Clark GM (2005). REporting recommendations for tumour MARKer prognostic studies (REMARK). Br J Cancer.

[35] Bremnes RM (2002). High-Throughput Tissue Microarray Analysis Used to Evaluate Biology and Prognostic Significance of the E-Cadherin Pathway in Non-Small-Cell Lung Cancer. J Clin Oncol.

