# Supplementary material for: Prognostic effect of intratumoral neutrophils across histological subtypes of non-small cell lung cancer
Source: Oncotarget. 2016 Sep 30;7(44):72184–96. doi: 10.18632/oncotarget.12360 (PMC5342153; doi:10.18632/oncotarget.12360)
Supplement: Supplementary file 1 [file oncotarget-07-72184-s001.pdf]

Prognostic effect of intratumoral neutrophils across histological subtypes of non-small cell lung cancer

SUPPLEMENTARY FIGURES AND TABLE

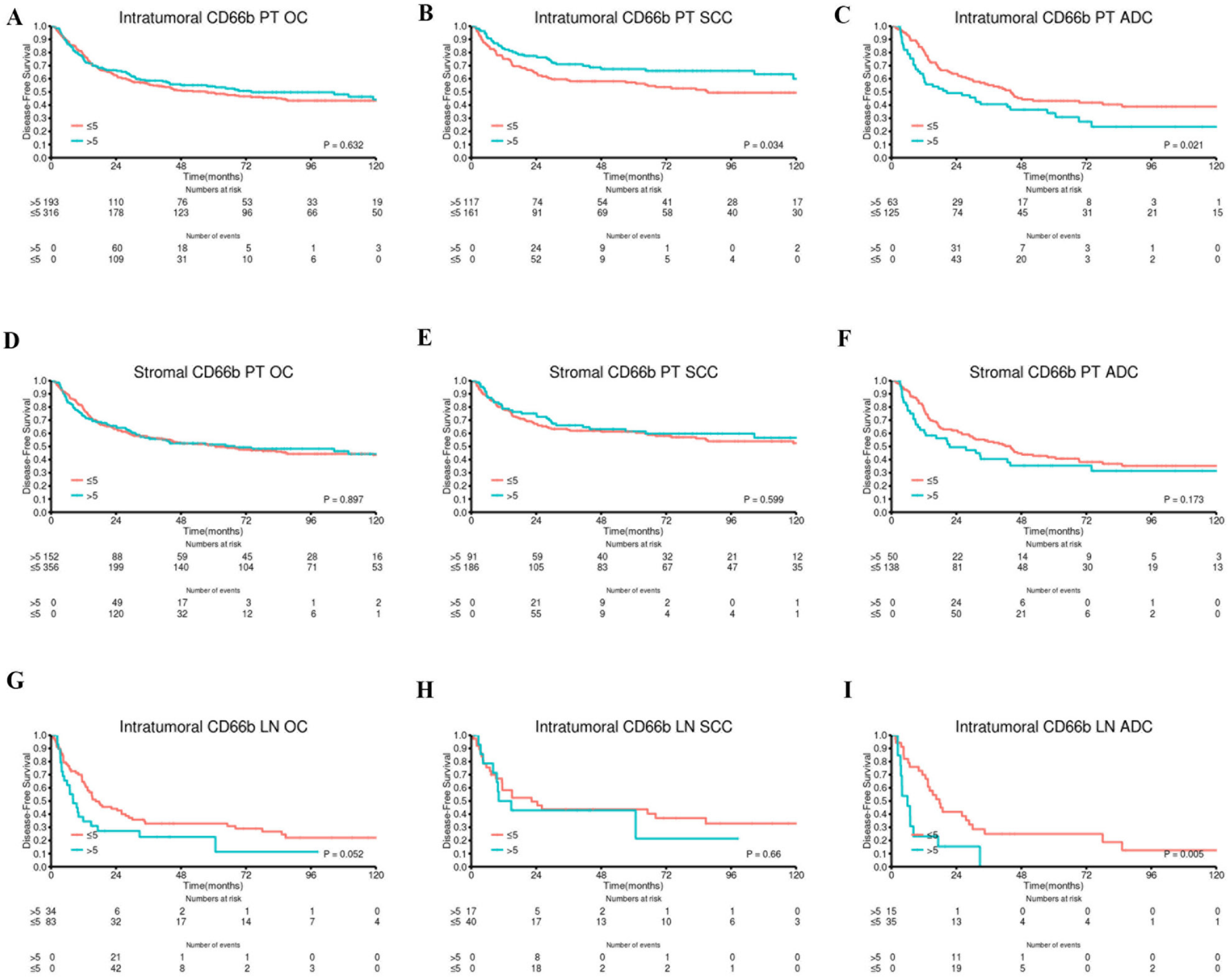

Supplementary Figure S1: Disease-free survival curves for A. Intratumoral CD66b in the overall cohort (OC) of primary tumors (PT); **B.** Intratumoral CD66b in squamous cell carcinomas (SCC) of PT; **C.** Intratumoral CD66b in adenocarcinomas (ADC) of PT; **D.** Stromal CD66b in the overall cohort of PT; **E.** Stromal CD66b in SCC of PT; **F.** Stromal CD66b in ADC of PT; **G.** Intratumoral CD66b in the overall cohort of LN+; **H.** Intratumoral CD66b in SCC of LN+; **I.** Intratumoral CD66b in ADC of LN+

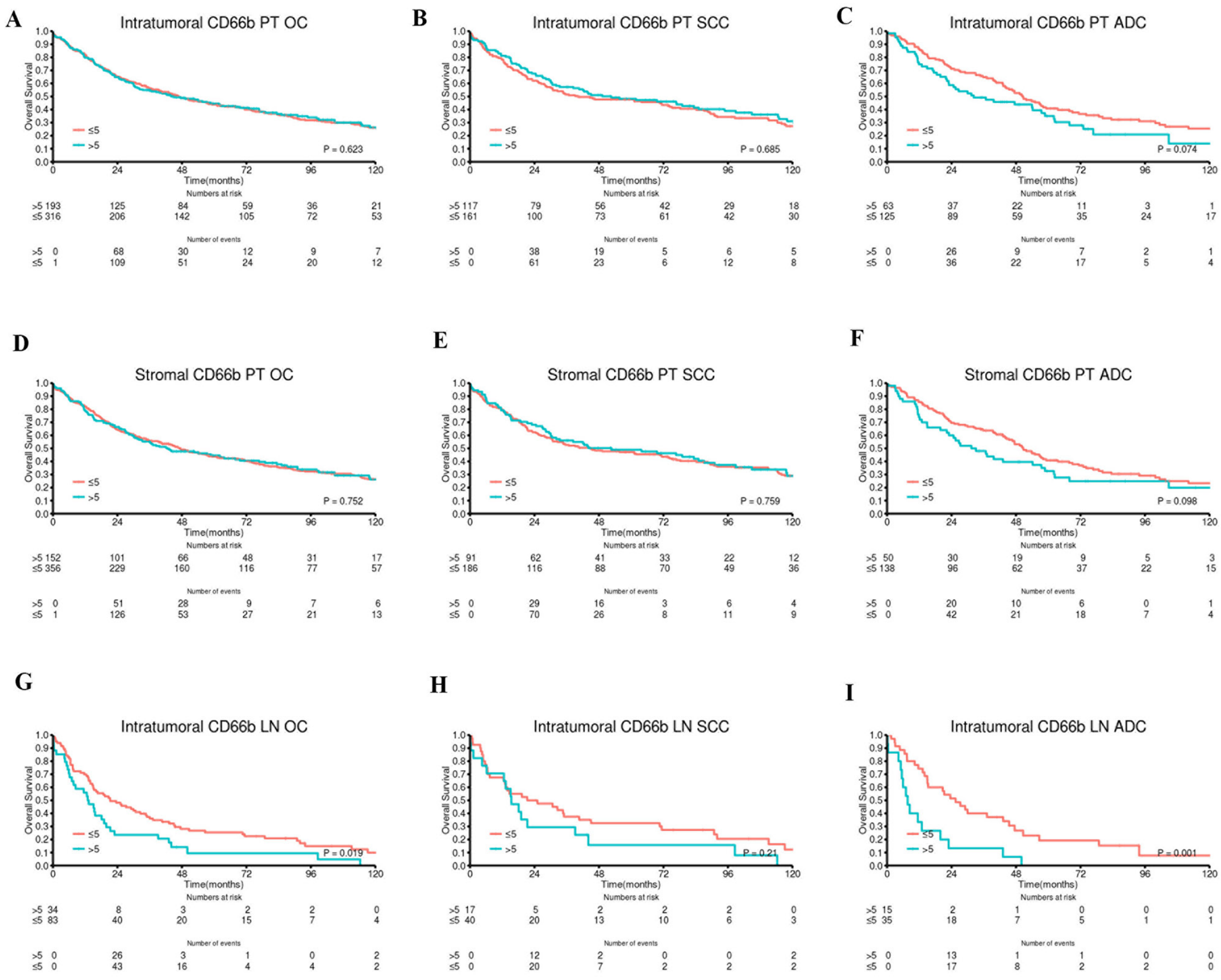

Supplementary Figure S2: Overall survival curves for A. Intratumoral CD66b in the overall cohort (OC) of primary tumors (PT); **B.** Intratumoral CD66b in squamous cell carcinomas (SCC) of PT; **C.** Intratumoral CD66b in adenocarcinomas (ADC) of PT; **D.** Stromal CD66b in the overall cohort of PT; **E.** Stromal CD66b in SCC of PT; **F.** Stromal CD66b in ADC of PT; **G.** Intratumoral CD66b in the overall cohort of LN+; **H.** Intratumoral CD66b in SCC of LN+; **I.** Intratumoral CD66b in ADC of LN+.

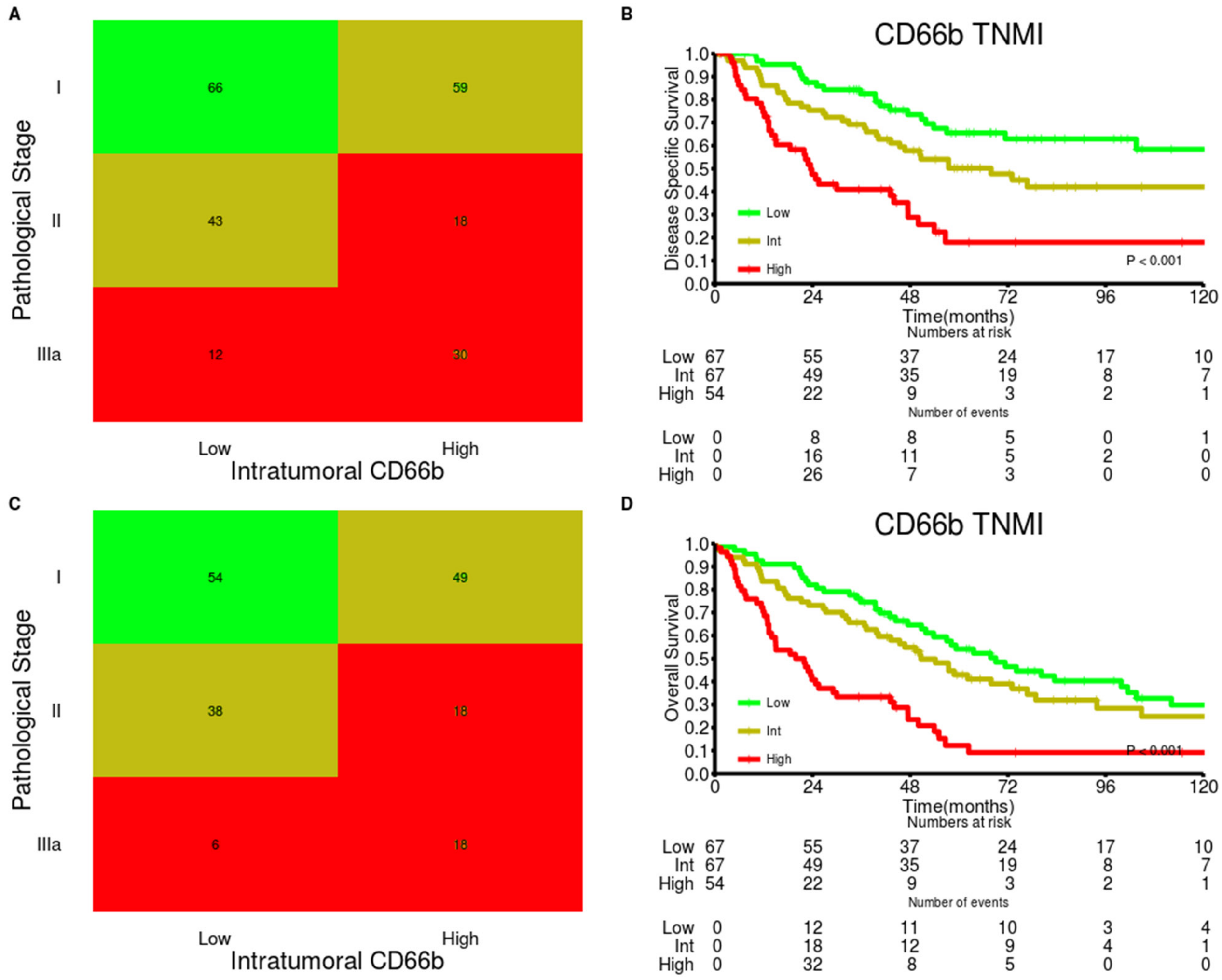

Supplementary Figure S3: CD66b TNM-I in ADC of NSCLC through DSS **A, B.** and OS **C, D.** endpoints. A, C) Distribution of ADC patients with similar survival in each pathological stages, and B, D) CD66b Immunoscore. (good prognosis: light green; intermediate prognosis: moss green; poor prognosis: red).

Supplementary Table S1: List of 104 tumor-associated markers (99 proteins and 5 microRNAs) investigated in our cohort.

See Supplementary File 1
