# Supplementary material for: Prognostic effect of intratumoral neutrophils across histological subtypes of non-small cell lung cancer
Source: Oncotarget. 2016 Sep 30;7(44):72184–96. doi: 10.18632/oncotarget.12360 (PMC5342153; doi:10.18632/oncotarget.12360)
Supplement: Supplementary file 2 [file oncotarget-07-72184-s002.docx]

Supplemental Table 1: List of 104 tumor-associated markers (99 proteins and 5 microRNAs) investigated in our cohort

|  | **Tumor** | **Stroma** | **Abbreviation/alternative name** |
| --- | --- | --- | --- |
| 1 | VEGF-A | VEGF-A | vascular endothelial growth factor A |
| 2 | VEGF-C | VEGF-C | vascular endothelial growth factor C |
| 3 | VEGF-D | VEGF-D | vascular endothelial growth factor D |
| 4 | VEGFR1 | VEGFR1 | vascular endothelial growth factor receptor 1 |
| 5 | VEGFR2 | VEGFR2 | vascular endothelial growth factor receptor 2 |
| 6 | VEGFR3 | VEGFR3 | vascular endothelial growth factor receptor 3 |
| 7 | PDGF-A | PDGF-A | platelet-derived growth factor A |
| 8 | PDGF-B | PDGF-B | platelet-derived growth factor B |
| 9 | PDGF-C | PDGF-C | platelet-derived growth factor C |
| 10 | PDGF-D | PDGF-D | platelet-derived growth factor D |
| 11 | PDGFR-α | PDGFR-α | platelet-derived growth factor receptor alpha |
| 12 | PDGFR-β | PDGFR-β | platelet-derived growth factor receptor beta |
| 13 | FGF2 | FGF2 | fibroblast growth factor 2 |
| 14 | FGFR1 | FGFR1 | fibroblast growth factor receptor 1 |
| 15 | Notch1 |  | neurogenic locus notch homolog protein 1 |
| 16 | Notch4 | Notch4 | neurogenic locus notch homolog protein 4 |
| 17 | Jag 1 | Jag 1 | jagged 1 |
| 18 | DLL4 | DLL4 | delta ligand 4 |
| 19 | SKB2 |  | SHK1 binding protein |
| 20 | ME2 |  | malic enzyme 2 |
| 21 | HIF1 | HIF1 | hypoxia inducible factor 1 |
| 22 | HIF2 | HIF2 | hypoxia inducible factor 2 |
| 23 | GLUT1 |  | glucose transporter 1 |
| 24 | LDH5 | LDH5 | lactate dehydrogenase A |
| 25 | CAIX | CAIX | carbonate dehydratase IX |
| 26 | Ang1 | Ang1 | angiopoietin 1 |
| 27 | Ang2 | Ang2 | angiopoietin 2 |
| 28 | Ang4 | Ang4 | angiopoietin 4 |
| 29 | Tie2 | Tie2 | angiopoietin 1 receptor |
| 30 | PHD1 |  | prolyl hydroxylase domain containing protein 1 |
| 31 | PHD2 |  | prolyl hydroxylase domain containing protein 2 |
| 32 | PHD3 |  | prolyl hydroxylase domain containing protein 3 |
| 33 | FIH |  | factor inhibiting HIF-1 |
| 34 | MMP2 | MMP2 | matrix metalloproteinase 2 |
| 35 | MMP7 |  | matrix metalloproteinase 7 |
| 36 | MMP9 | MMP9 | matrix metalloproteinase 9 |
| 37 | IntA5B1 |  | Integrin alpha 5 beta 1 |
| 38 | MET-k |  | hepatocyte growth factor receptor |
| 39 | MET-g |  | phospho-Met (Tyr1234/1235) |
| 40 | MCT1 | MCT1 | monocarboxylate transporter 1 |
| 41 | MCT2 | MCT2 | monocarboxylate transporter 2 |
| 42 | MCT3 | MCT3 | monocarboxylate transporter 3 |
| 43 | MCT4 | MCT4 | monocarboxylate transporter 4 |
| 44 | PGC1-α |  | peroxisome proliferator activated receptor gamma coactivator 1 |
| 45 |  | agma | alpha smooth muscle Actin |
| 46 | COL4A3 | COL4A3 | collagen type IV alpha 3 |
| 47 | End |  | endostain |
| 48 | TSP1 |  | thrombospondin 1 |
| 49 | D240 | D240 | podoplanin |
| 50 | CD34 | CD34 |  |
| 51 |  | CD31 |  |
| 52 | CD4 | CD4 |  |
| 53 | CD45 | CD45 |  |
| 54 | CD8 | CD8 |  |
| 55 | CSF1R | CSF1R | colony stimulating factor 1 receptor |
| 56 | MCSF | MCSF | macrophage colony-stimulating factor |
| 57 | CD68 | CD68 |  |
| 58 | CD20 | CD20 |  |
| 59 | CD56 | CD56 |  |
| 60 | CD1A | CD1A |  |
| 61 | CD3 | CD3 |  |
| 62 | CD138 | CD138 |  |
| 63 |  | CD117 |  |
| 64 | CXCL16 | CXCL16 | chemokine CXC motif ligand 16 |
| 65 | CXCR6 | CXCR6 | chemokine CXC motif receptor 6 |
| 66 | FOXP3 | FOXP3 | forkhead box protein P3 |
| 67 | tAkt | tAkt | phospho-Akt (Thr308) |
| 68 | sAkt | sAkt | phospho-Akt (Ser473) |
| 69 | bAkt | bAkt | Akt2 |
| 70 | cAkt | cAkt | Akt3 |
| 71 | pi3k | pi3k | phosphatidylinositol 3 kinase |
| 72 | PTEN | PTEN | phosphatase and tensin homolog |
| 73 | pHer1 |  | phospho- human epidermal growth factor receptor 1 |
| 74 | pHer2 |  | phospho- human epidermal growth factor receptor 2 |
| 75 | pHer3 |  | phospho- human epidermal growth factor receptor 3 |
| 76 | Her4 |  | phospho- human epidermal growth factor receptor 4 |
| 77 | NfKB | NfKB | nuclear factor kappa-B |
| 78 | Vimentin | Vimentin |  |
| 79 | Par6 | Par6 | partitioning-defective protein 6 |
| 80 | APKc |  | protein kinase C zeta type |
| 81 | E-cadherin |  | epithelial calcium dependant adhesion protein |
| 82 | Fascin |  |  |
| 83 | TGF-β |  | transforming growth factor beta |
| 84 | Bad |  | Bcl-2 associated death promotor |
| 85 |  | Masson | masson's trichrome |
| 86 | COX-2 | COX-2 | cyclooxygenase-2 |
| 87 | IGF1 | IGF1 | insulin like growth factor 1 |
| 88 | IGFBP2 | IGFBP2 | insulin like growth factor binding protein 2 |
| 89 | Dicer |  | double-strand-specific ribonuclease |
| 90 | Drosha |  | double stranded RNA specific endoribonuclease 3 |
| 91 | ERK3_N |  | extracellular signal-regulated kinase(nucleus) |
| 92 | ERK3_C |  | extracellular signal-regulated kinase(cytoplasm) |
| 93 |  | ERK3 | extracellular signal-regulated kinase |
| 94 | p27 |  | cyclin-dependent kinase inhibitor 1B |
| 95 | p21 |  | cyclin-dependent kinase inhibitor 1A |
| 96 | p16 |  | cyclin-dependent kinase inhibitor 2A |
| 97 | FOXO1A | FOXO1A | forkhead box protein O1A |
| 98 | pFOXO1A | pFOXO1A | Phospho- forkhead box protein O1A (phospho-S256) |
| 99 | Ki67 | Ki67 |  |
| 100 | miR182b |  | micro RNA 182b |
| 101 | miR21 | miR21 | micro RNA 21 |
| 102 | miR210 | miR210 | micro RNA 210 |
| 103 | miR126 |  | micro RNA 126 |
| 104 | miR155 | miR155 | micro RNA 155 |
